# The safety and efficacy of dorsal penile nerve block for premature ejaculation: A systematic review and meta-analysis

**DOI:** 10.1097/MD.0000000000016479

**Published:** 2019-07-26

**Authors:** Song Sun, Liang Han, Yufeng Li, Xudong Yu, Binghao Bao, Hong Zhou, Ziqi Gong

**Affiliations:** a Department of Surgery, Beijing Changping Hospital of Traditional Chinese Medicine; b Department of Andrology, Fangshan Hospital, Beijing University of Chinese Medicine, Fangshan District, Chinan; c Department of Andrology, Dongzhimen Hospital, Dongcheng District, Hai Yun Cang on the 5th ZIP, Beijing, China.

**Keywords:** dorsal penile nerve block, premature ejaculation, protocol, systematic review

## Abstract

**Background:**

Premature ejaculation is a common sexual dysfunction disease in adult males. There are many clinical trials shown that dorsal penile nerve block can prolong the ejaculation latency to a certain extent in the vagina. In this study, we aim to use a meta-analysis to evaluate the efficacy and safety of dorsal penile nerve block for premature ejaculation.

**Methods and analysis:**

We will search for PubMed, Cochrane Library, AMED, EMbase, WorldSciNet, Nature, Science online and China Journal Full-text Database (CNKI), China Biomedical Literature CD-ROM Database (CBM), and related randomized controlled trials included in the China Resources Database. The time is limited from the construction of the library to February 2019. The quality of the included RCTs will be evaluated with the risk of bias (ROB) tool and evidence will be evaluated by GRADE. Data analysis will be used the special software like RevMan (version 5.3) and EndNote X7.

**Results:**

The results of this meta-analysis will be submitted to a peer-reviewed journal for publication.

**Ethics and dissemination:**

This systematic review will evaluate the efficacy and safety of dorsal penile nerve block for premature ejaculation. Because all of the data used in this systematic review and meta-analysis has been published, this review does not require ethical approval. Furthermore, all data will be analyzed anonymously during the review process Trial.

**Trial registration number:**

PROSPERO CRD42019119691

## 1 Introduction

Premature ejaculation (PE), one typical kind of male sexual dysfunction, does not have an internationally acknowledged uniform standard so far. Numerous scholars believe that PE means ejaculation occurs within one minute during the sexuality with sex partner after inserting into the vagina, and this symptom lasts for at least 6 months.^[1,2]^ PE, also known as early ejaculation, rapid ejaculation or rapid climax, is the most common male sexual dysfunction. Surveys in the United Kingdom and the United States have shown that the prevalence of PE is 20% to 30%.^[3,4]^ According to the time when the symptom of PE occurs, this disease could be divided into two types which we named preliminary premature ejaculation and acquired premature ejaculation.^[5]^ Men who suffer from PE are more likely to reflect lower sexual function and satisfaction than men without PE, and this defection also reflects in the overall quality of life. Besides, it has been reported that the satisfaction of a partner's sexual relationship increasingly decreases with the progression of the disease. And the patients are more vulnerable to suffering from mental illness such as anxiety and depression, which also causes obvious pain to them.^[6]^

The etiology of PE is complex, and its pathogenesis is still unclear. We traditionally believe that this disease is mostly related to psychological factors.^[7–9]^ Recent researches have focused on the combined effects of psychological and organic factors. It is generally believed that persistent unhealthy psychological factors may aggravate potential abnormal organic factors, above which lead to the occurrence of premature ejaculation.^[10,11]^ Increasing studies have shown that the excitability of the penile dorsal nerve in premature ejaculation patients is higher than that in normal people.^[12]^ Therefore, during sexual intercourse, the ejaculation latency (IELT) and the ejaculation reflex arc short ejaculation threshold are low, which lead to premature ejaculation during sexual intercourse. Yet, precise therapy of PE has not been formulated, present therapeutic schedule mainly include psychological therapy, behavioral therapy, drug therapy and Chinese medicine.^[13,14]^ The therapies we illustrate above could be used alone or in combination, and relevant literature have reported certain clinical effects. However, there are still many problems such as large side effects, high recurrence rate, and long-term efficacy uncertainty, which we need to concentrate on.^[15–18]^

The clinical application of dorsal penile nerve block has opened up a new therapeutic approach for the treatment of premature ejaculation.^[19]^ As early as 1993, Tullii reported the use of dorsal penile nerve block in treating premature ejaculation. The theory of this method for treating premature ejaculation is that the stimulation of the penis skin and glans is transmitted through the dorsal nerve of the penis, and the erection of the penis is initiated. The method blocks the free back nerve by surgery, thereby reducing the sensitivity of the sensory nerve of the penis head, increasing the penile sensory threshold, and therefore prolonging the ejaculation latency.^[20]^ In recent years, this treatment has been tried in some countries, and its efficacy has been recognized to some extent. The treatment method has the characteristics of simple operation, safety and effectiveness, short treatment period and little pain, and has been highly paid attention to by the domestic and international urology and male science circles.^[21,22]^

After a preliminary search analysis of the database, we found that the amount of randomized controlled trials of dorsal penile nerve block for PE has been increasing.^[23]^ Previous clinical trials have shown that this surgical method has a good short-term effect on prolonging the ejaculation latency and the ability to control the ejaculation of PE patients. However, due to the size and number of clinical centers, the current level of evidence-based medical evidence is still at a lower level of evidence. Therefore, we are aimed at using meta-analysis to evaluate the efficacy and safety of dorsal penile nerve block in the treatment of PE, and providing evidence for its clinical application.

## 2 Methods

This systematic review protocol has been registered on PROSPERO as CRD42019119691. (https://www.crd.york.ac.uk/prospero/display_record.php?RecordID=119691). The protocol follows the Cochrane Handbook for Systematic Reviews of Interventions and the Preferred Reporting Items for Systematic Reviews and Meta-Analysis Protocol (PRISMA-P) statement guidelines. We will describe the changes in our full review if needed.

### 2.1 Inclusion criteria

#### 2.1.1 Types of studies

This study will include all randomized controlled trials of all dorsal penile nerve block for PE. For the included randomized controlled trials, investigators need to explicitly report randomized methods, surgical procedures and parameters, as well as diagnostic criteria and efficacy evaluation indicators based. No restriction in whether it has been published. The experiment is limited to humans. Language is limited to Chinese and English.

#### 2.1.2 Types of participants

The included cases are adult male patients over 20 years old who have diagnosed PE. (Refer to the Diagnostic Criteria for PE for the revised edition of the Diagnostic and Statistical Manual of Mental Disorders issued by the International Society of Medical Sciences and the American Psychiatric Association.) The course of suffering from PE is ≥6 months. Excluding prostatitis, seminal vesiculitis, urinary tract infections, diabetes, high blood pressure, and other diseases. The patient's sexual partner is stationary. The group was well balanced when enrolled. The region, nation, ethnic, and sources are not limited.

#### 2.1.3 Types of interventions

##### 2.1.3.1 Experimental interventions

The experimental intervention is the treatment of PE with the dorsal penile nerve block. Separate surgery or surgery combined with medication for PE will be included. The secondary dorsal penile nerve block will be limited.

##### 2.1.3.2 Control interventions

As for the control interventions, we will accept no treatment as a blank control. Or use medication alone as a control intervention.

#### 2.1.4 Types of outcome measures

##### 2.1.4.1 Primary outcomes

The primary outcome measures will be assessed by using changes in intravaginal ejaculation latency Time (IELT) before and after treatment in both groups. Efficacy judgment setting: IELT >5 min is set to be effective after treatment; IELT 3 to 5 min is improved after treatment; IELT <3 min is invalid after treatment. Effective = markedly effective + improved. Effective rate = (number of effective patients + number of patients improved)/total number of patients×100%.

##### 2.1.4.2 Secondary outcomes

Secondary outcome measures will include:

1. Arabic index of premature ejaculation (AIPE) [Chinese randomized controlled trials will use a more appropriate Chinese premature ejaculation assessment tool: China PE Questionnaire (CIPE)];
2. International erectile function scores (IIEF-5) Changes before and after treatment;
3. Comparison of the effectiveness of the groups and the incidence of postoperative complications and adverse events.

### 2.2 Data source

Database Search: PubMed, Cochrane Library, AMED, EMbase, World SciNet, Nature, Science online and China Journal Full-text Database (CNKI), China Biomedical Literature CD-ROM Database (CBM), China Resources Database. The clinical literature on the treatment of PE from dorsal penile nerve block published in domestic and foreign biomedical journals from the establishment of the library to February 2019 was searched. Based on the standards of the Cochrane Collaboration Workbook of the International Evidence-Based Medicine Center, a manual and computer-based approach is used to conduct related literature searches. The search terms include: dorsal penile nerve block, penile nerve selective severance, premature ejaculation, early ejaculation, rapid ejaculation rapid climax and Sexual dysfunction. The search term in the Chinese database is the translation of the above word. The complete PubMed search strategy is summarized in Table 1.

**Table 1:**
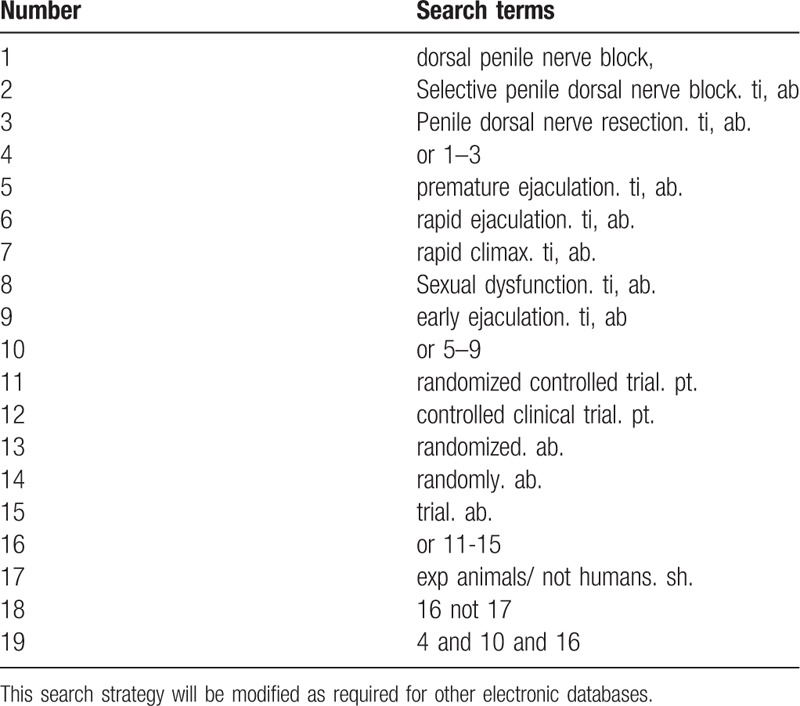
Search strategy used in PubMed database.

### 2.3 Data collection and analysis

#### 2.3.1 Selection of studies

Two investigators used EndnoteX7 software to conduct a preliminary assessment of the title and abstract of each document in the database based on the established criteria for inclusion in the study to select eligible studies. After a preliminary assessment, the full text of the selected literature would be evaluated, and the uncontrolled study, no randomization, inconsistent evaluation criteria, and similar data would be excluded. Any differences in screening that occurred during the screening study would be discussed in order to get consensus, if it still cannot be resolved, then the third author would be intervened.

#### 2.3.2 Data extraction and management

**Figure 1:**
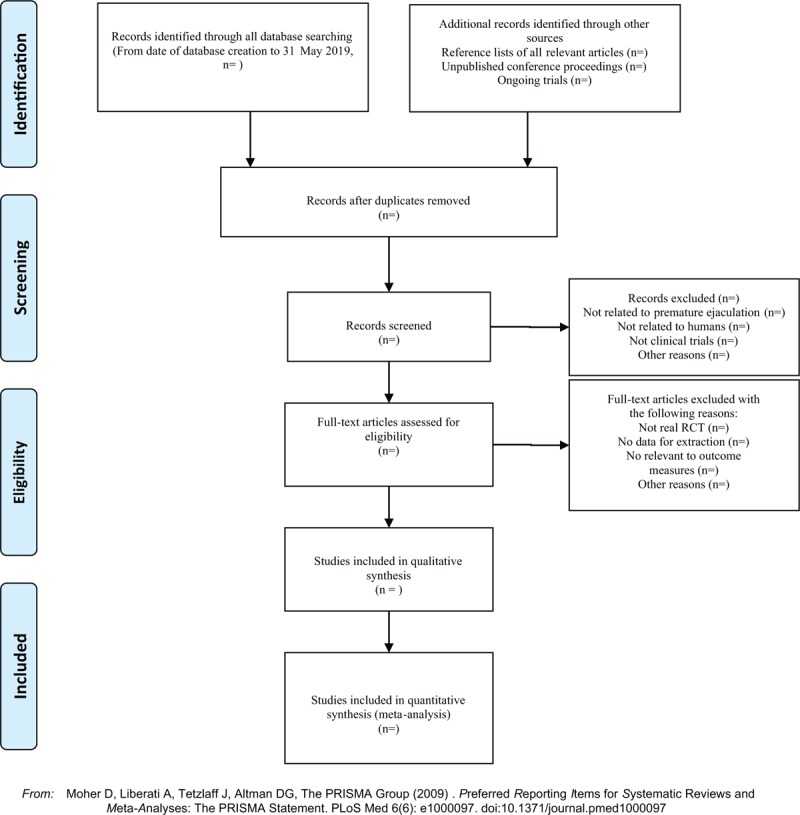
The PRISMA flow chart.

Two investigators independently extracted information from the included literature. The extracted content includes research design, random hiding and blinding, basic information of the included cases, intervention methods, observation indicators and test results of the treatment group and the control group. The extracted literature data will be filled in a unified data statistics table. For studies that provide baseline and post-treatment data, we will estimate the change values by the method recommended by Cochrane. The primary selection process is shown in a PRISMA flow chart (Fig. 1)

#### 2.3.3 Assessment of risk of bias in included studies

Two investigators will independently evaluate the methodological quality of the included literature by using the Cobrane Collaboration's ROB tool which includes whether the random method is correct, whether blinding is used, whether it is hidden, whether it is lost or quit, whether it uses Intent-To-Treat (ITT) analysis, whether the data results are accurate, and other risks of bias. According to the relevant standards in the Cochrane Intervention System Evaluation Manual, it will be divided into low risk, high risk, and unclear.

#### 2.3.4 Dealing with missing data

In the event of data loss during the screening and extraction of literature data, primarily, we will actively investigate the cause of data loss. Then we will contact the experimental research author by telephone, mail, etc. to achieve the purpose of supplementing the missing data. If the lost data cannot be retrieved, we will only extract and analyze the useful data, besides, we will indicate the situation.

#### 2.3.5 Statistical analysis

The meta-analysis in this study will use Rev Man5.3 and Stata13.0 statistical software. Heterogeneity tests will be used for the included experimental studies. The numerical variable will be expressed as the normalized mean difference (SMD) with a confidence interval (CI) of 95%. The heterogeneity of each pairwise comparison will be tested by chi-square test (test level α = 0.1). If there is no heterogeneity, a fixed effect model will be used. If there is significant heterogeneity between a set of studies, we will use a random effects model (REM) for meta-analysis. We will explore the reasons for the existence of heterogeneity from various aspects such as the characteristics of the subjects and the degree of variation of the interventions. The source of heterogeneity is further determined by means of sensitivity analysis.

#### 2.3.6 Assessment of heterogeneity

If there is significant heterogeneity between a group of studies, we will explore the reasons for the heterogeneity from multiple aspects such as the characteristics of the subjects and the degree of variation in the interventions. Sensitivity analysis or subgroup analysis is performed as necessary to explain heterogeneity.

#### 2.3.7 Assessment of publication bias

If a result of a meta-analysis contains more than 10 articles and above, we will use Rev Man5.3 software to map and analyze the forest map, funnel plot. Quantitative methods such as Begg testing and Egger testing will be used to help assess publication bias in the application.

#### 2.3.8 Grading the quality of evidence

The quality of evidence for the main outcomes will also be assessed with the GRADE approach. The evaluation included bias risk; heterogeneity; indirectness; imprecision; publication bias. And each level of evidence will be made “very low,” “low,” “moderate,” or “high” judgment.

## 3 Discussion

At present, there are many treatments for PE, but due to the complexity of the pathogenesis of PE, the efficacy still could not be satisfactory. Studies have shown that SSRI drug intervention can prolong the ejaculation latency of PE patients and improve their sexual function scores.^[24,25]^ However, clinical reports have reported that there are many problems such as many side effects, high recurrence rate, and long-term efficacy uncertainty, thus, there is currently no drug that can continue to significantly ameliorate all symptoms of PE patients.^[16,26]^ In recent years, domestic and international studies have shown that the main cause of PE is hypersensitivity of the penis head and excessive excitation of the ejaculation center. The biosensory threshold of the penile head of PE patients is significantly lower than that of the healthy control group, and the latency of somatosensory induction potential induced by penile head stimulation is significantly shorter than that of the healthy control group.^[27,28]^ Some scholars have suggested that the cause of PE may be closely related to the decrease of penile sensory threshold and increased nerve excitability.^[29]^ Therefore, the application of partial resection of the dorsal sensory nerve endings of the penis can reduce the “pulsation” of the nerve impulses that are transmitted to the dorsal nerve of the penis, thereby reducing the sensitivity of the penis head, prolonging the ejaculation latency, and improving the quality of life of PE patients.

Recently, clinical trials on the treatment of PE with dorsal penile nerve block have shown an increasing trend, and the efficacy of the report has been recognized to some extent. However, due to the instability of postoperative complications and the incomplete evaluation mechanism of postoperative long-term efficacy, there is still much doubt in the academic community about the effectiveness and safety of this treatment.^[30]^ Therefore, we hope to explore the effectiveness and safety of penile dorsal nerve blockade in the treatment of premature ejaculation through the meta-analysis. Its results could provide a possible ranking for the treatment of PE with dorsal penile nerve block. We also hope that the results will provide clinicians with the best option for treating PE. In addition, due to the limited number of relevant high-quality studies and the small sample size included, the strength of the conclusion's argument is limited. Therefore, we hope that more large-scale, high-quality randomized controlled trials will be needed in the future, and long-term follow-up mechanisms will be established. At the same time, it is necessary to improve the quality of the original research and conduct high-quality multi-center randomized controlled trials to explore the clinical efficacy of dorsal penile nerve block in the treatment of PE, which could make the conclusion more objective and reasonable.

## Author contributions

**Conceptualization:** Yufeng Li.

**Data curation:** Liang Han, Binghao Bao, Ziqi Gong.

**Funding acquisition:** Song Sun.

**Resources:** Song Sun.

**Supervision:** Song Sun.

**Writing – original draft:** Xudong Yu, Hong Zhou.

**Writing – review & editing:** Hong Zhou, Ziqi Gong.
